# Exploring the potential of dietary factors and plant extracts as chemopreventive agents in oral squamous cell carcinoma treatment

**DOI:** 10.3389/froh.2023.1246873

**Published:** 2023-10-04

**Authors:** Madhav Kumar, Abhimanyu Kumar Jha

**Affiliations:** Department of Biotechnology, Sharda School of Engineering and Technology, Sharda University, Greater Noida, India

**Keywords:** dietary factors, plant extracts, chemopreventive properties, oral squamous cell carcinoma, phytochemicals

## Abstract

Oral cancer, particularly oral squamous cell carcinoma (OSCC), is a prevalent malignancy having a significant fatality rate worldwide. Despite advancements in conventional treatment modalities, the overall survival rate for OSCC remains low. Therefore, there is a critical need to explore alternative therapeutic approaches that can improve patient outcomes. This review focuses on the potential of dietary factors and plant extracts as chemopreventive agents in treating oral cancer. These compounds possess diverse biological functions encompassing a range of attributes, such as antioxidative, anti-inflammatory, and anticancer capabilities. By targeting multiple cellular pathways involved in carcinogenesis, they possess the capacity to hinder tumor growth and development, promote programmed cell death, and impede the progression of oral cancer. Signaling pathways targeted by natural compounds that have been included in this review include Akt/mTOR/NF-κB signaling, Hippo-Tafazzin signaling pathway, notch signaling pathway, mitochondrial pathway, and Sonic Hedgehog pathway.

## 1. Introduction

It is widely acknowledged that cancer is one of the primary contributors to mortality worldwide, and in contrast with non-communicable disease (NCDs), cancer is currently the second most common cause of death worldwide (1). By 2030, the burden of cancer is expected to triple despite the enormous amount of research that is now being done (2). Plant-derived phytochemicals are essential for drug development and cancer therapy, controlling the molecular pathways associated with the growth and advancement of cancer (3). The main objective of this review is to explain what we know till now about the dietary factors and plant extract, along with their chemopreventive properties. Cancer chemoprevention uses synthetic or natural compounds to control, slow, or reverse cancer occurrence, including preventing precancerous lesions (4). Dietary compounds are considered suitable for cancer prevention because they have lower toxicity levels when compared to regular drugs. Phytochemicals act as chemopreventive agents, offering potential benefits for preventing all stages of carcinogenesis. Bioactive phytochemicals found in certain foods have the capacity to modify the activity of oncogenes and genes that suppress tumor formation, potentially preventing cancer initiation and progression (5). Oral cancer (OC) is a highly widespread malignancy globally and is the subtype of head and neck cancer. OC is caused by a variety of changes in genes, which appear moderately over time along with the tumor's capacity to circumvent the host immune system (6). The oral cavity, which includes lip, tongue, gums, base of the mouth, cheeks, and palate, experiences aberrant cancerous tissue growth. Approximately 32% of instances arise in the buccal mucosa, 11% in the lower lip, 22% in the tongue, 11% in the palate, 5% in the floor of the mouth, 3% in the gingiva, 8% in the vestibule, and 5% in the alveolus. Oral cancer rates are increasing in certain countries, including Sri Lanka, Pakistan, India, Taiwan, and Bangladesh, constituting about 25% of newly diagnosed cases. This type of cancer is predominantly observed in men (7). More than 95% of cases of oral cancer are identified as carcinomas, often known as **oral squamous cell carcinomas** (OSCC) (8). Squamous cell carcinoma originates from epithelial tissue dysplasia with morphological changes like basal cell hyperplasia, nuclear hyperchromatism, and increased nuclear-cytoplasmic ratio in cancer patients (9). Of all neck and head cancer, 38% is caused by OSCC (10). In addition to having high rates of morbidity and death worldwide, **OSCC** also exhibits treatment resistance. Despite improvements in treatments, like radiation, surgery, and chemotherapy, the survival rate is 5 years in individuals with OSCC and has remained at or around 50% for a number of decades (11). The 2020 GLOBOCAN data show that there were 377,713 newly reported cases of oral cancer and 177,757 deaths annually. With regard to India, GLOBOCAN data show 75,290 deaths and 135,929 new cases per year. In India, OC is responsible for 50%–70% of all cancer-related deaths and is most prevalent in Asian countries (12). Smoking and drinking can have synergistic effects, as tobacco smoke contains over 4,000 carcinogens, including methoxymethylfurfural, nicotine, methanol, and arsenic. Alcohol activates procarcinogens and acts like a solvent for harmful carcinogens to enter body cells. Once tumors reach a finite size, pain develops and medical assistance is needed (7). Unhealthy diets contribute to cancer and other diseases, while adopting a healthy lifestyle reduces cancer risk. International organizations advocate consuming specific vegetables, fruits, and grains linked to lower tumor and cancer risk. Certain foods contain phytochemicals, non-nutritional compounds with health benefits. These chemicals protect plants from external threats and can provide similar protection for humans, like neutralizing free radicals in the body (13).

Numerous dietary factors are bioactive agents with cancer-preventive and therapeutic properties. Ursolic acid (UA) induces caspase-dependent apoptosis and modulates biological biomarkers, together with downregulating PI3K/mTOR/Akt and activating ERK, p38, and NF-κB signaling (14). Epigallocatechin gallate (EGCG) shows promise as a potential innovative therapeutic substance for cisplatin-resistant cancer as well as human oral cancer. EGCG triggers cellular autophagy and apoptosis, simultaneously suppressing the expression of MDR1 in CAR cells (15). Resveratrol from red grape skin causes apoptosis in tongue squamous cell carcinoma cells by decreasing cell viability, enhancing apoptotic processes, and inhibiting cell migration via the mitochondrial pathway and transcription factors, which induces endothelial to mesenchymal transition (EMT) (16). *Syzygium cumini* (SC) extract hinders oral cancer cell growth, prompting cell death by accumulating ROS, making it a potential preventive measure for OSCC (17). Pomegranate (*Punica granatum*) impacts cytoskeleton dynamics, cancer cell anoikis, and chemotaxis, suggesting antimetastatic potential through extracellular matrix changes. Furthermore, it acts on pro-inflammatory and pro-angiogenic molecules involved in cancer metastasis, offering a versatile approach to inhibit tumor spread (18).

Curcumin, a polyphenol present in the rhizome of turmeric, shows potential activity as a cancer treatment, focusing on pathways like NF-κB and MAPK (19). Its antioxidant, analgesic, anti-inflammatory, and anticarcinogenic properties make it a promising treatment. Quercetin, a polyphenol found in various plants and fruits, has been found to possess anticancer properties because of its potential to inhibit enzymes involved in carcinogen activation and attach to cellular receptors and proteins (20). I*n vitro* studies have shown that quercetin can induce cell death in oral cancer; inhibit the NF-κB, MMP-2, and MMP-9 signaling pathways; and prevent migration and invasion. Isothiocyanates (ITCs), made through hydrolysis of glucosinolates in cruciferous vegetables, are well known for their antioxidant, antibacterial, and anticancerous properties (21). Sulforaphane (SFN) is effective in various cancers by affecting enzymes, apoptosis, cell cycle, microRNAs, oxidative stress, HDAC inhibition, and angiogenesis (22). Lycopene (LYC), an antioxidant found in tomatoes, grapefruit, pomegranates, and watermelons, has been shown to have anti-fibrotic effects, protecting cells from reactive oxygen species damage (23). Lycopene administration increases E-cadherin expression in oral cancer cells, reducing EMT and promoting apoptosis through the PI3K/AKT/mTOR signaling pathway (24).

## 2. Oral cancer types

### 2.1. Oral squamous cell carcinoma

OSCC is a general and dominant oral cancer type. A defective p53 gene is reported to have a significant role in checkpoint controls and apoptotic processes in around 50% of OSCC. OSCC typically manifests and frequently originates on the tongue, lips, and surfaces within the oral cavity (25).

### 2.2. Lymphoma

Lymphomas represent the second most common form of primary head and neck malignancies and are malignant tumors originating from the immune system. Lymphomas are divided into two types: non-Hodgkin’s lymphoma (NHL) and another is Hodgkin’s lymphoma (HL). NHL’s incidence is increasing in several countries, with a range of 1%–17% in the head and neck area (26).

### 2.3. Oral melanoma

Malignant melanoma is a rare and aggressive form of cancer, primarily affecting the gingiva, hard palate, and maxillary alveolar mucosa. Males are more susceptible to developing this form, with only 3%–5% of all cutaneous cancers being malignant. The 5-year survival rate is 15%–38%, and the prognosis for this condition is generally poor compared to cutaneous malignancies highlighting the increased challenges and severity associated with this specific type of cancer. Oral melanoma commonly affects various areas within the oral cavity including the floor of the mouth, tongue, lips, and buccal mucosa (25). Among these, the buccal gingiva and palate are frequently affected regions by oral melanoma.

## 3. Oral squamous cell carcinoma development–related risk factors

### 3.1. Alcohol

Alcohol is a notable risk factor for oral cancer development due to its alteration of mucosa morphology, lipid dissolution, DNA synthesis destruction, DNA repair enzyme interference, and impaired salivary gland function. These modifications increase the possibility of mouth cancer (27).

### 3.2. Tobacco

According to the International Agency for Research on Cancer (IARC), tobacco smoking is classified as the type I carcinogen including smokeless tobacco in oral cavity (28). Data provided by the IARC suggest that more than 60 different carcinogens are found in cigarette smoke (29).

### 3.3. Betel quid

Betel quid (BQ) is toxicologically harmful to human health. BQ usage is an increasing health concern in Asia and among certain migrant populations in western nations (30). BQ is a chewing combination containing areca nut (AN), slaked lime, betel leaf, and regional flavors. It is a psychostimulant that is addictive and carcinogenic. BQ compounds are classified as category I carcinogens by the IARC, with increased risks for oral and pharyngeal cancers and potential oral malignant diseases. Animal studies show that BQ can cause mouth and pharynx malignancies (30).

**Figure 1:**
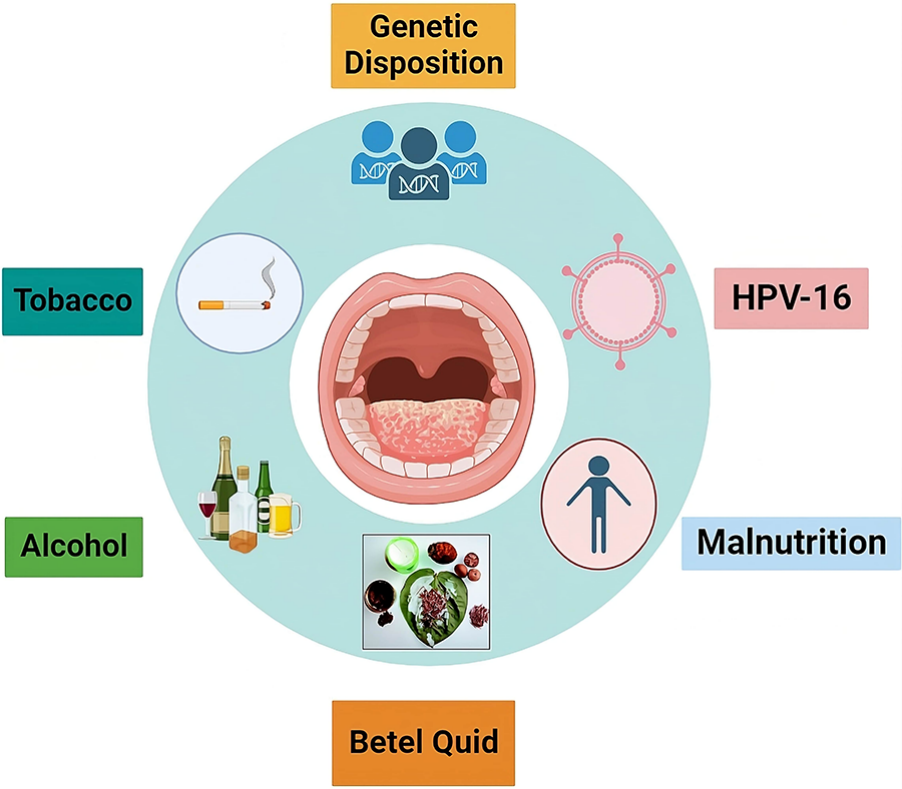
Summary of risk factors associated along with OSCC. These risk factors are mainly responsible for causing oral cancer.

In addition, a number of putative risk elements responsible for oral cancer development include genetic predispositions, chronic irritability, poor dental hygiene, occupational exposure, malnutrition, low fruit and vegetable diets, and viral infections. HPV-16 (Figure 1), a human papilloma virus, has been identified as an etiological agent for squamous cell carcinoma in younger individuals, particularly around the tongue and tonsillar region. This raises concerns about HPV-negative squamous cell carcinoma (29). Figure 1 shows the potential risk factors responsible for oral squamous cell carcinoma.

## 4. Rationale for exploring dietary factors and plant extracts as chemopreventive agents

Dietary factors and plant extract have been a valuable source of anticancer agents in various fields. Despite numerous latest and expensive treatments, the results are not significantly refreshing (1). Although their efficacy, chemotherapy medications can also have negative effects on normal cells, such as myelosuppression, alopecia, mucositis, nausea, and vomiting. They have also been linked to multidrug resistance (MDR), a dangerous condition that accounts for approximately 90% death rate of the cancer patients receiving chemotherapy (31). MDR development has been seen in many patients making chemotherapy less impactful. Anticancer drugs have side effects like cardiotoxicity, ototoxicity, and cognitive impairment, compromising adherence (32). Adverse effects like hair loss, fatigue, kidney damage, gastrointestinal problems also contribute to the side effects of treatment (32).

Dietary factors are now studied as the potential cytotoxic agents, leading to innovative strategies to tackle different types of cancer and accelerate the clinical research. Dietary factors significant molecular diversity and unique biofunctionality make them crucial for drug discovery. Dietary factors offer higher efficacy and safety profile because of their extraordinary molecular properties. Dietary factors have the ability to provide intracellular signals that start processes that kill cancer cells (1).

## 5. Important signaling pathway and their dysregulation in oral squamous cell carcinoma

### 5.1. PI3K/Akt/mTOR/NF-κB signaling pathway

The development of OSCC is correlated with Akt/mTOR/NF-κB pathway dysregulation as well as with constitutive NF-κB activation. Numerous physiological functions, including synthesis of protein, autophagy, regulation of cell cycle, metabolism of glycogen, synthesis of fatty acid, food absorption, and nuclear protein organization, are impacted by the AKT/mTOR signaling pathway. In addition, it affects other signaling pathways including NF-κB, ERK, and JAK/STAT, as well as impacting cancer-related traits like proliferation, survival, angiogenesis, invasion, migration, and apoptosis (34).

Protein translation, apoptosis, and survival of tumor cells are all significantly influenced by the PI3K/AKT signaling pathway. Receptor tyrosine kinases stimulate PI3K, causing the enzyme to become allosterically active and the regulatory subunit to become tyrosine phosphorylated. Phosphatidylinositol-4,5-bi-phosphate (PIP2) phosphorylation at 3′-hydroxyl group by PI3K results in the production of PIP3 in response to external stimuli. AKT, a member of the mTOR complex, is phosphorylated by PIP3 and mTOR and gets activated. PTEN, a widely recognized regulator of the PI3K/AKT signaling pathway, suppresses the growth of tumors by lowering the cyclin D1 level and causing G1 cell cycle arrest (35). The activity of signaling protein AKT was found to be significantly increased due to the somatic mutation in cancer cells. This increased expression of AKT activity has a profound impact on downstream cell process, which leads to cell death. The mutation in AKT results in suppression of two Bad and Bax proapoptotic proteins. A cascading effect generated due to Bad inhibition results in the activation of an anti-apoptotic protein, which leads to inhibition of apoptosis.

The FOXO-1 (forkhead box protein O1) is another important participant in the apoptotic process that AKT also targets. The transcription factor FOXO-1 regulates two proapoptotic genes, Bim (Bcl-2-like protein 11) and FasL (Fas ligand). These are the genes that are responsible for triggering the cell death signals, which promote apoptosis (36–38). AKT primarily targets mTOR protein kinase downstream. It accomplishes this by tuberous sclerosis complex (TSC)-2 phosphorylation, repressing its expression and blocking the initiation of RHEB (Ras homolog enriched in brain). Consequently, RHEB-Guanosine-5′-triphosphate (GTP) is able to initiate mTORC1 (mammalian target of rapamycin complex 1) by attaching to it (39).

**Figure 2:**
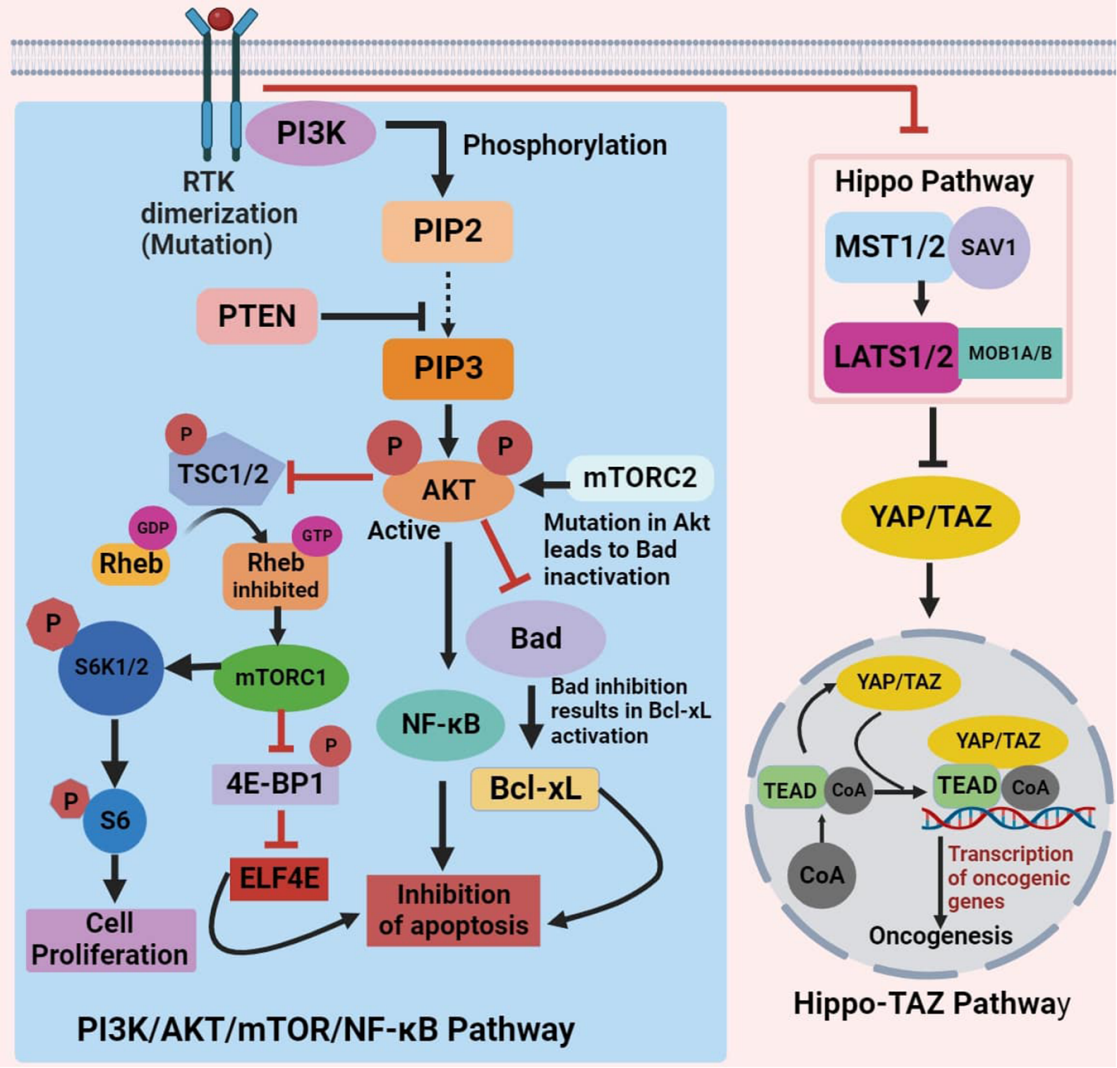
Schematic of PI3K/AKT/mTOR/NF-κB and hippo-TAZ signaling pathway dysregulation inhibiting apoptosis and inducing oncogenesis in oral cancer.

In addition, mTORC1 activation triggers the eIF4e (eukaryotic translation initiation factor 4) complex, leading progression of cell cycle and tumor growth while inhibiting cell death. To fully activate AKT, mTORC2 phosphorylates residues of serine in its region of C-terminal, specifically, Ser474 in AKT2, Ser472 in AKT3, and Ser473 in AKT1. The activity of both mTORC1 and mTORC2 is regulated by the accessibility of these active sites, which are under the control of mTOR-associated proteins (40). Various proteins interact with mTOR complexes, impacting cell processes. mTORC1 activates S6 and regulates cell functions and translation through 4E-BP1 (Figure 2). Meanwhile, PI3K and ribosomes activate mTORC2 and aid its functioning (34). In tissues taken from the hard palate, alveolar ridge, and gingiva of OSCC, active forms of mTOR and AKT proteins were abundant. This indicates that the initiation of mTOR/AKT is linked to the progression of OSCC (35).

### 5.2. Hippo-Tafazzin signaling pathway

Tafazzin (TAZ) transcriptional activity relies on nuclear recruitment, facilitating binding to various transcription factors, notably the TEAD family. This transcriptional regulation leads to cell proliferation, survival, and migration, contributing to TAZ's pro-tumorigenic functions. Multiple signaling events, including Hippo pathway activation, restrict TAZ from entering the nucleus by inducing phosphorylation on conserved serine residues, forcing to their blockade into the cytoplasm. Mechanical cues and G protein–coupled receptor signals also influence TAZ localization through both Hippo pathway–dependent and Hippo pathway–independent mechanisms (41). The Hippo signaling pathway, a well-preserved signaling mechanism, triggers the translocation of TAZ, into the nucleus upon activation. There, it ties up with the TEA domain family of transcription factor, leading to alterations in various gene expression linked to migration, survival, and proliferation of cells (42) (Figure 2). Stimulation of the Hippo-TAZ pathway in squamous cell carcinoma of tongue (TSCC) cells fosters proliferation, migration, and invasion, while also suppressing apoptosis (41, 43]).

### 5.3. Notch signaling pathway

The Notch signaling pathway, a juxtacrine signaling system, activates the genes that are responsible for cell survival, angiogenesis, and proliferation. It has been observed to have high expression levels in various cancers, including OSCC, contributing to tumor growth and spread (44) The Notch pathway becomes active when ligands interact with Notch receptors in neighboring cells. In mammals, there are four transmembrane receptors, Notch 1, Notch 2, Notch 3, and Notch 4, and these receptors interact with ligands including Jagged 1 and 2, Delta-like 1, 2, and 4 that participate in this juxtacrine signaling pathway (45).

### 5.4. The mitochondrial pathway

The mitochondrial signaling pathway of apoptosis is crucial for controlling cell death against different stimuli. Bcl-2 family members induce mitochondrial outer membrane permeability transition. Programmed cell death, also known as apoptosis, can be triggered through two primary pathways: the intrinsic (involving the mitochondria) and the extrinsic (involving death receptors) pathways. Focusing on apoptosis has been proposed as a potential approach for eradicating cancer cells. In the mitochondrial pathway, the proapoptotic protein Bax, which is a member of the Bcl-2 family, triggers cytochrome c secretion in cytosol (16). Cytochrome c ties up with Apaf-1 to form a complex that creates the apoptosome while combining with caspase-9. This initiates caspase-3 and caspase-9, leading to apoptosis through the cleavage of important cellular proteins. Caspase-3 acts as a crucial executor by cleaving cellular proteins, including PARP. DNA fragmentation is regulated by CAD and its inhibitor (ICAD). Apoptotic signal causes caspase-3 to cleave ICAD, activating CAD for DNA fragmentation (16).

### 5.5. Sonic Hedgehog pathway

The signaling pathway Sonic Hedgehog (SHh) is crucial for development of humans, and improper control of this system can lead to the growth of malignancies. SHh are dormant in tissues of adults. These pathways are usually carcinogenic when they exhibit abnormal activation in adult tissues (46). One of the most crucial routes in vertebrates, the SHh system controls a variety of activities throughout development of embryo, including development of oral mucosa and skin (47). When the Sonic Hedgehog protein (SHh) binds to the transmembrane receptor patched (PTCH1), it leads to the release of Smoothened (Smo), which is also a transmembrane protein. This event triggers an intracellular sequence that leads to the movement of a transcription factor known as Gli-1 (Glioma-Associated Oncogene Homolog 1) into the cell's nucleus. Gli-1 activates gene transcription in this nucleus that is connected to the cell division and cell cycle. PTCH-1 (Patched 1), GLI-1, CCND1, oncogene B-cell leukemia 2 (BCL2), WNT-1, EGFR, β-catenin, and family members of transforming growth factor β are notable genes impacted by this process (48). It was discovered that the expression of SHh in dysplastic oral epithelium was much higher. Overexpression of transcription factor Gli-1 and SHh was observed by 10-fold and fivefold, respectively, indicating that alteration in the Sonic Hedgehog pathway leads to the formation of tumor in oral cancer (49).

## 6. Chemopreventive properties of dietary factors and plant extracts in oral cancer

**Figure 3:**
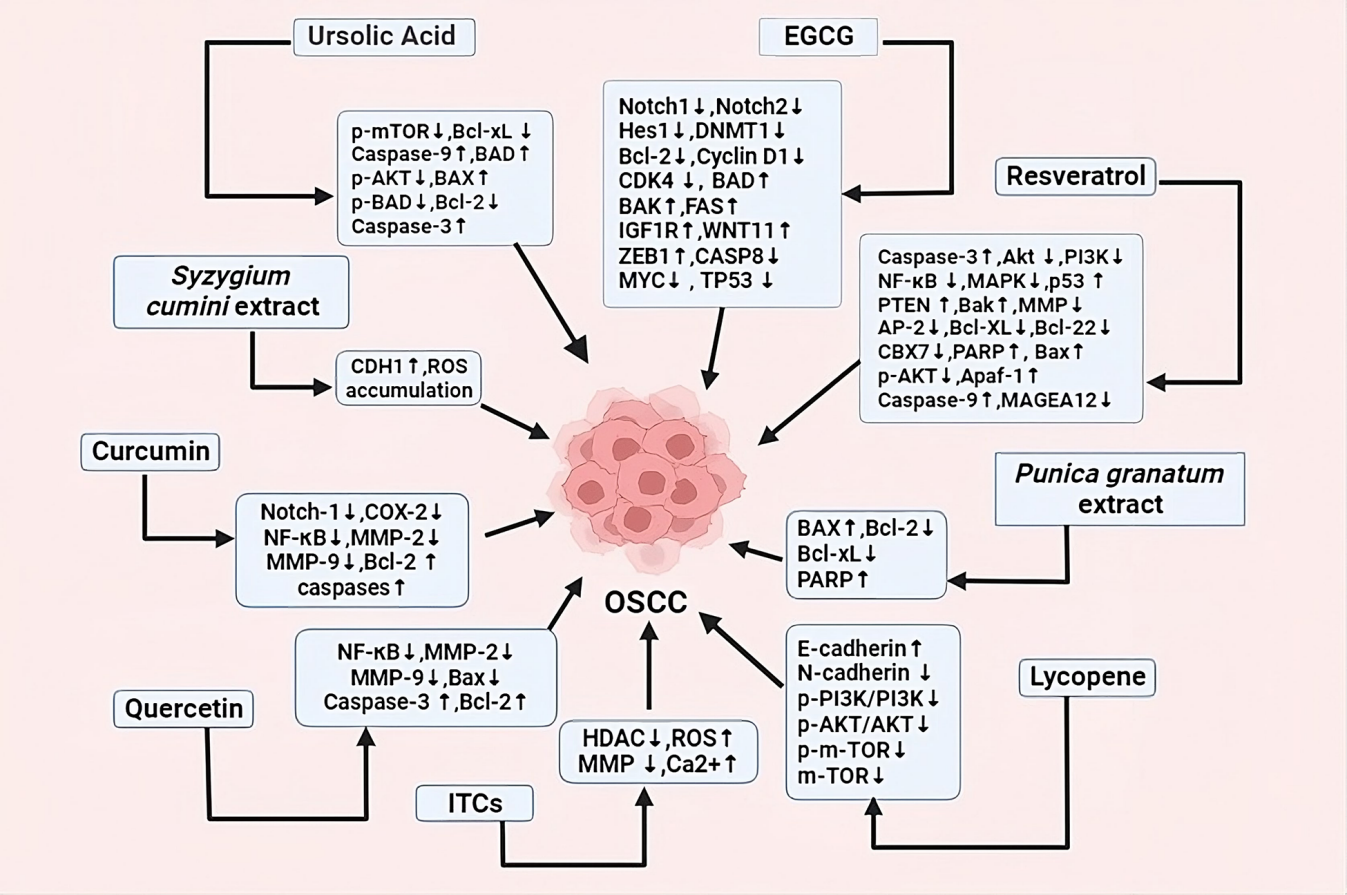
Dietary factors and plant extracts showing anticancerous properties by regulating different signaling pathways and genes, causing programmed cell death, cell cycle arrest, autophagy, decreasing EMT etc.

Plants are the main sources of bioactive phytochemicals, which are valuable medicinal ingredients in the pharmaceutical industry. These phytochemicals are effective in treating various diseases, including inflammation, cancer, Alzheimer's, coronary heart disease, and infectious diseases. There are numerous ways to categorize phytochemicals, including polyphenols, terpenes, alkaloids, saponins, and flavonoids (50). More than half of novel medications come from plants or use synthetic substances with phytochemicals as their pharmacophore (51). Phenolic compounds, including phenolic acids and carotenoids, are found in various plants. Coffee, blueberries, cherries, and cinnamon contain hydroxycinnamic acid, while hydroxybenzoic acid is found in only a few plants (52). Overproduction of oxidants in the body causes cancer, while phytochemicals in dietary fruits, vegetables, and grains protect against cancer development. Lifestyle changes can prevent over two-thirds of cancers and contribute to 35% of cancer mortality (53). Figure 3 shows role of different dietary factors as a chemopreventive agent in treating oral cancer by targeting different signaling pathways and regulating different genes in oral cancer treatment.

### 6.1. Dietary factors and plant extracts showing anticancerous properties by targeting different signaling pathways in oral squamous cell carcinoma

Dietary factors and plant extract show promising effect against oral cancer. They achieve this by targeting different pathways and influencing different genes. Dietary factors and plant extract activate or deactivate these pathways and genes directly or indirectly showing their ability in reducing cancer cell growth or inducing apoptosis. Different mechanisms of action of these dietary factors and plant extracts are mentioned in this review in detail, and their summary has been included in Table 1 along with their references.

**Table 1:** Showing different mechanism of action by dietary factors and plant extract.

Dietary factors and plant extracts	Mechanism of action		References
	Signaling pathway	Apoptotic pathway	
1. Ursolic acid	Akt/mTOR/NF-*κ*B	Caspase-9↑, BAD↑, BAX↑ p-AKT↓, Caspase-3↑	Lin et al. (14) Chen et al. (62)
2. EGCG	Notch signaling, Hippo-Tafazzin	Notch1↓, Notch2↓ BAD↑, BAK↑, FAS↑	Li et al. (42) Wei et al. (54) Irimie et al. (65)
3. Resveratrol	Mitochondrial pathway	Caspase-3↑, p53↑, CBX7↓, PARP↑, Caspase-9↑	Chen et al. (55)
4. *Syzygium cumini* extract	ROS pathway	Inducing ROS, CDH1↑	Ezhilarasan et al. (17) Yang and Lian (56)
5. *Punica granatum* extract	Mitochondrial pathway	Bcl-2↓, Bcl-xL↓, PARP↑, BAX↑	Peng et al. (57) Lee et al. (73)
6. Curcumin	PKB/Akt/NF-κB, MAPK	Caspases↑, Notch-1↓, NF-κB↓, Bcl-2↑	Zhen et al. (19), Liao et al. (58)
7. Quercetin	Mitochondrial signaling pathways	Caspase-3↑, Bcl-2↑, Bax↓, NF-κB↓	Ma et al. (59) Singh et al. (60)
8. ITCs	Mitochondrial pathway	HDAC↓, ROS↑ MMP↓, Ca2+↑	Krishnan et al. (22), Mitsiogianni et al. (61)
9. Lycopene	PI3K/AKT/mTOR	E-cadherin↑, N-cadherin ↓, p-PI3K/PI3K↓, p-mTOR↓ mTOR↓	Wang et al. (24)

#### 6.1.1. Ursolic acid

The plant triterpenoid UA has been found to have anticancer properties and to inhibit cell growth in a variety of malignant tumors. UA generally contained in the stem bark, leaves, and fruit peels of several plants and foods, such as peppermint, lavender, basil, apples, oregano, cranberries, and so on (62). PI3K/Akt/mTOR, ERK1/2 MAPK and Stat5/Akt, p53, cyclo-oxygenase-2, and Bcl-2 are some of the signaling pathways that UA uses to induce apoptosis in the case of OSCC (14). By employing a concentration-dependent approach, ursolic acid obstructs the phosphorylation of mTOR, Akt, and NF-κB. In addition, it decreases the levels of p38 and phosphorylated ERK (14). In oral cancer cell lines, especially Ca922**,** UA induces apoptosis by caspase-dependent cell death. Flow cytometry evaluation showed that utilizing a concentration-dependent way, UA enhanced the proportion of apoptotic cells. In UA-treated Ca922 cells, it had been seen that there is a cleavage in caspase-7, caspase-9, and PARP, which confirms apoptosis. The comet test demonstrated that treatment with UA caused an increase in DNA strand breaks in Ca922 cells using a concentration-dependent way, which further supported the notion that UA causes apoptosis (14).

#### 6.1.2. (-)-Epigallocatechin gallate

EGCG is the most prevalent and physiologically active catechin compound found in green tea and has been rigorously explored for its potential to treat cancer (63). Green tea stimulates some of the detoxifying enzymes like quinone reductase and glutathione-s-transferase, which protects against carcinogenesis. Extracts from green tea may be able to stop precancerous lesions from turning malignant into OSCC. Overall, it was shown that patients receiving green tea extracts had a higher clinical response rate (8). EGCG has potential anticancer activity through various mechanisms, including apoptosis induction, signal transduction regulation, oxidative stress inhibition, angiogenesis inhibition, arresting cell cycle, and reducing cancer cell proliferation. By modifying a number of signaling pathways such as Wnt, JAK/STAT, and Notch, the development of cancer is very efficiently prevented by EGCG (64).

The biological responses of OSCC cell lines CAL27 and SCC15 to EGCG were shown to be concentration and time dependent. The levels of the proteins TAZ, LATS1, MOB1, and JNK were also lowered by EGCG. The impact of EGCG on CAL27 cells was diminished by TAZ overexpression. In addition, CAL27 cells’ proliferation, migration, and invasion were dramatically reduced, and their rate of apoptosis was elevated when EGCG and simvastatin were combined. EGCG influences the Hippo-TAZ signaling system, which controls TSCC migration, invasion, apoptosis, and proliferation (42).

EGCG plays an important role in inducing apoptosis and inhibiting cancer in tongue cells. *In vivo* experiments show that it inhibits tumor growth in KRas-mutated mice by partially suppressing the Notch signaling pathway (54). EGCG treatment in a dose-dependent approach on tongue cancer cells like CAL-27 and SCC9 inhibited proliferation and a large number of apoptotic cells (42). Apoptotic marker levels increased and anti-apoptotic proteins such as Bcl-2 decreased after the treatment (54). It directly hinders the DNMT1 molecule by forming hydrogen bonds and indirectly through COMT (catechol-O-methyl transferase)-mediated methylation. This process leads to a reduction in the methyl donor SAM and the formation of SAH, a potential demethylating agent (64).

The intrinsic apoptosis pathway is regulated by the proapoptotic BCL-2 family proteins BAD and BAK, with BAK playing a crucial role in this process, which EGCG particularly activates in SSC-4 cells. When compared to oral epithelium, BCL-2 expression is lower in OSCC. The EGCG treatment activates FAS, BAK, BAD, ZEB1, WNT11, and IGFIR while it obstructs TP53, MYC, and CASP8. These findings indicate that EGCG triggers death in tumor cells by programmed cell death and autophagy, and has a high therapeutic potential to become drug for OSCC patients (65).

EGCG downregulates Notch1, Notch2, and Hes1 at mRNA as well as protein level, while Cyclin D1 and CDK4 expression decreased. This suggests that EGCG suppresses the Notch signaling pathway, resulting in hampering proliferation of cell and also causes tongue cancer cell apoptosis (54). EGCG treatment on caspase 3 wild-type oral cancer cell lines has been demonstrated to cause a progressive reduction in mitochondrial activity down to a negligible level; however, apoptosis was not seen in caspase 3 null cells (8).

HSC-3 (Human Squamous Cell Carcinoma) cells displayed a decrease in cell growth and were halted at the G1 cell cycle phase following exposure to EGCG. High dosages of EGCG have a strong inhibitory effect on the OSCC cells (scc-25) development and proliferation, and they also impede DNA synthesis (11).

#### 6.1.3. Resveratrol

The main source of resveratrol is red grapes, berries, peanuts, and some food products. It is a polyphenolic compound having potent anti-inflammatory, antioxidant, cardioprotective, and antitumor qualities (16). Through inhibiting a number of important regulators of cell survival pathways, including AP-2, NF-κB, PI3K/Akt, and MAPK, and activating genes that are responsible for suppressing tumor-like p53 and PTEN (phosphatase and tensin homolog), resveratrol can inhibit cell proliferation, induce programmed cell death, and cell cycle transition disruption at the G1-S phase. By suppressing Akt and increasing AMPK signaling in CAR cells of human oral cancer cells, resveratrol caused autophagy. Aberrant enhancement of the Akt/mTOR pathway promotes tumor cell growth, proliferation, survival, and resistance to programmed cell death induced by drugs. During resveratrol-induced apoptosis in CAR cells, there was DNA condensation and fragmentation. Resveratrol activates AMPK by phosphorylating Ulk1 at Ser317, and in turn promotes cell autophagy, which also aids in the apoptosis of CAR cells (66). Resveratrol is responsible for the downregulation of MMP, which also activated Bak and Bax. Resveratrol inhibited Bcl-XL and Bcl-22 and releases cytochrome c from the mitochondria and activated ICAD and PARP, which induces apoptosis (16). This suggests that in OSCC cells, resveratrol triggers apoptosis through activating the mitochondrial route and caspase cascades.

Resveratrol inhibits OSCC cell proliferation by enhancing apoptosis via inhibiting the CBX7 protein. The CBX7 gene (chromobox homolog 7), which is found on chromosome 22q13.1, has encoded a chromobox protein and may have a separate vital role in the initiation and progression of certain tumors. CBX7 and p-AKT expression levels in cells considerably reduced following resveratrol therapy; however, the expression of p16 dramatically increased when compared with the control. These results suggested that resveratrol could prevent the CBX7 pathway from being activated. Depending on the concentration, resveratrol increased the expression of cleaved caspase-3 and cleaved PARP while simultaneously reducing the levels of total PARP (t-PARP) expression. Resveratrol also enhanced the histone DNA amount utilizing a concentration-dependent pathway and enhanced caspase-3 and caspase-9 (55). By inhibiting CBX7/Akt and activating p16 cascades, resveratrol impedes the growth in OSCC cells and promotes apoptosis.

RCP (Rab coupling protein) is a key factor in HNSCC, impacting patient progression and overall survival. RCP induces Zeb1 expression, which then obstructs oral cancer invasion induced by RCP. Zeb1 also enhances expression of MT1-MMP to promote invasion, which is mediated by the β1 integrin/EGFR/β-catenin pathway. Resveratrol suppresses the RCP-induced expression of Zeb1 by disrupting the recycling of β1 integrin endosomes and the activation of EGFR. This inhibition results in the suppression of RCP-induced oral cancer invasion (67).

By using a Western blotting test, resveratrol administration also caused caspase activation in CAL27 cells. After treatment with resveratrol, there was an increase in apoptosis protease activating factor 1 (Apaf-1), and activation was observed in pro-caspase 9, pro-caspase 3, cleaved caspase 3, PARP, and ICAD (67).

Resveratrol can suppress the signaling pathway MAGEA12/Akt against OSCC, a pathway which is involved in the invasion, migration, and proliferation of tumor cells. Overexpression of MAGEA12 showed a substantial increase in cell feasibility compared to the control group. After 48 h of treatment with resveratrol, a notable reduction in the MAGEA12/Akt pathway was observed, with the resveratrol IC50 measured at 50 μM. However, it is worth noting that the inhibitory effect of resveratrol was somewhat diminished in cells overexpressing MAGEA12/Ark proteins. This observation suggests that the downregulation of the MAGEA12/Ark pathway may play a role in the anticancer effects of resveratrol (68).

#### 6.1.4. *Syzygium cumini* extract

SC also called **Jambul** is frequently used in traditional medicine mainly in India to cure a vast range of illnesses, including diabetes and obesity. The plant's seeds contain a variety of phytoconstituents, including the alkaloid jambosine and the glycosides jambolin and antimellin (17).

SC seed kernel methanolic extract can induce growth inhibitory effects in human OSCC cells through a dose-dependent way. The seeds, containing high amounts of tannins, terpenes, and simple phenols, were studied for their potential cytotoxicity. SC induces cytotoxicity in OSCC through a dose-dependent way. SC treatments accumulate ROS intracellularly in OSCC cells. The highest level of ROS expression was seen in cells that are treated with a SC dose (17).

SC treatments significantly changed OSCC cell morphology. Lower concentration of SC caused early apoptosis, while higher concentration leads to late apoptosis. Membrane damage in late apoptotic cells is supported by the presence of red cells in EB. Treatment with SC leads to increase in apoptotic cell population through a concentration-dependent way (17).

#### 6.1.5. *Punica granatum* extract

*Punica granatum*, commonly called **Pomegranate**, prevents a wide range of malignancies (18, 69). Pomegranate abundantly contains polyphenols (70, 71). POMx is the polyphenolic extract from pomegranate that shows effective anticancer activity in oral cancer cells focusing on its impact on mitochondrial functioning. POMx inhibits mitochondrial activity, which causes oral cancer cells to proliferate less and undergo apoptosis. POMx causes programmed cell death in oral cancer cell lines through the mitochondrial pathway, while it does not have an impact on normal cells. It also disrupts the membrane potential of mitochondria and generates mitochondrial superoxide, downregulating antioxidant gene expression. POMx also induces DNA damage in mitochondria and nucleus, suggesting that POMx provides antiproliferation and apoptosis through mitochondrial impairment mechanisms (57).

Incubation of normal cell and oral cancer cell lines with POMx at various doses resulted in dual staining patterns for annexin V/7AAD. A high number of apoptotic oral cancer cells have been seen in POMx-incubated cells. There is an enhancement in BAX and PARP, while downregulation in Bcl-2 and Bcl-xL. POMx might be able to inter-regulate apoptosis and autophagy, which is confirmed by the decrease in autophagy detected by Acridine Orange (AO) in oral cancer cell lines induced by POMx (57).

#### 6.1.6. Curcumin

Curcumin, a polyphenolic molecule extracted from the rhizome of the turmeric plant, gained widespread recognition for its potential as a chemopreventive and chemotherapeutic treatment for cancer. *In vitro* and *in vivo* studies suggested that curcumin might cure a few cancer types, including oral, prostate, lung, and breast cancer. Curcumin is responsible for targeting a wide variety of molecular targets, such as PKB/Akt/NF-κB and MAPK. Curcumin's anticancer properties also regulate EGFR expression and EGFR downstream signaling pathways (19). Curcumin exhibits a wide range of advantageous properties, including antioxidative, analgesic, anti-inflammatory, anticarcinogenic, antiseptic, chemopreventive, chemotherapeutic, antitumor, anti-infective (against bacteria, fungi, and viruses), and anti-platelet effects (72). The curcumin-induced downregulation of Notch-1, which would result in the decrease of NF-κB and the suppression of invasion and cell growth, may be a successful strategy in combating OSCC (58). By inhibiting COX-2 (cyclo-oxygenase-2) and cytokines, curcumin has anti-inflammatory properties. It also inhibits MMP-2 and MMP-9, prevents tumor metastasis, and regulates the Bcl-2 and caspases activation, which causes cancer cells to enter the programmed cell death (73).

#### 6.1.7. Quercetin

Quercetin, a polyphonic substance, is present in many dietary fruits and plants like dill, lovage, capers, onions, cilantro, cranberries, and apples Quercetin has anticancer properties that are attributable to multiple cell signaling systems, and it has the capacity to obstruct enzymes involved in carcinogen initiation. Furthermore, quercetin has anticancerous properties through attaching with cellular receptors and proteins (20). Quercetin causes death in oral cancer SAS cell line *in vitro* through mitochondria signaling pathways and endoplasmic reticulum (ER) stress (59). By suppressing NF-κB and matrix metalloproteinase-2 and 9 pathways in SAS human OCCs, and through a cell cycle halt coupled with mitochondria-mediated programmed cell death in SCC-25 cells, quercetin prevents migration and invasion *in vitro* (60). The injection of quercetin with a dosage of 40 μM strongly triggered programmed cell death and displayed PARP cleavage in many cell lines of oral cancer such as SCC1483, SCC25, and SCC QLL1. In addition, a caspase 3 activity experiment demonstrated that quercetin's ability to induce apoptosis was dependent on caspase 3 (74). Quercetin causes programmed cell death, suppresses Bax expression, activates expression of Casp3 and Bcl-2, and reversal of MDR mediated by gene-encoded P-glycoprotein in KB/VCR cells by obstructing P-glycoprotein expression (75).

#### 6.1.8. Isothiocyanates

ITCs are a category of substances produced by the myrosinase (enzymatic hydrolysis) of GLs (glucosinolates), a sulfur-containing substance found in cruciferous vegetables. Cruciferous vegetables like cabbage, broccoli, mustard, horseradish, and radishes could be an excellent choice since they contain chemicals such as isothiocyanates that have strong antioxidant, antibacterial, and anticancer properties. BITC (Benzyl ITC), AITC (allyl ITC), I3C (indole3-carbinol), PEITC (phenylethyl ITC), sulforaphane, sulforaphene, iberin, and erucin are the main ITCs present in cruciferous vegetables (21).

SFN is effective in various cancers, by affecting cell cycle, programmed cell death, microRNAs, oxidative stress, inhibiting HDAC, enzyme regulation, and angiogenesis. SFN inhibited cell growth in oral cancer and activity of HDAC, which resulted in cell cycle arrest and death. ROS production increased, MMP levels dropped, and the apoptotic pathway was activated (22).

Oral squamous carcinoma cell growth was reported to be effectively inhibited by PEITC (0.5–5M) by arresting cell cycle and mitochondrial-dependent programmed cell death as a result of ROS generation and Ca2+ buildup (61).

#### 6.1.9. Lycopene

LYC is a well-known carotenoid antioxidant found in abundance in tomatoes, pomegranates, pink grapefruit, and watermelons. *In vitro* studies on human fibroblast activity and rat hepatic fibrosis both point to lycopene's potential anti-fibrotic effects. Cells damaged by free radicals brought on by reactive oxygen species can be protected from this damage by lycopene. It inhibits the NO and H2O2 formation, prevents NO-mediated membrane destruction, and death in cells. It also lowers the possibility of lymphocyte DNA to oxidative damage (23). Lycopene demonstrated to possess anticancerous effects on various cancer types like prostrate, breast, lung, colon, and stomach cancers. In OC cells, LYC administration raised E-cadherin expression and lowered the N-cadherin expression, while substantially and in a dose-dependent manner, it decreases the p-PI3K/PI3K, p-AKT/AKT, and p-mTOR/mTOR expression ratio. This suggested that by stimulating the PI3K/AKT/mTOR pathway, LYC may decrease the EMT process and promote programmed cell death in OSCC (24).

## 7. Conclusion

Dietary factors and plant extracts have the capability to act as chemopreventive agents in oral cancer treatment. Dietary factors and plant extracts, which has been discussed in detail like ursolic acid, EGCG, resveratrol, *Syzygium cumini* extract, *Punica granatum* extract, curcumin, quercetin, ITCs, and lycopene, show anticancer effects by causing apoptosis through different signaling pathways. By targeting multiple cellular pathways involved in carcinogenesis, these compounds have the ability to stop the tumor growth, induce apoptosis, and prevent the development and progression of oral cancer. It has been shown that these dietary factors possess diverse properties like antioxidant, anti-inflammatory, anticancer activities. The utilization of dietary factors in the form of plant extracts offers several benefits like bioavailability, synergistic effects, and reduced side effects compared to conventional therapies. Dietary factors utilize different pathways like PI3K/Akt/mTOR/NF-κB signaling, Hippo-TAZ signaling pathway, Notch signaling pathway, mitochondrial pathway, and Sonic Hedgehog pathway in oral cancer.

Overall, the study of dietary factors and plant extracts as chemopreventive agents in treating oral cancer is a promising field of study. There is a chance to transform the area of cancer therapies and give people suffering from this horrible illness fresh hope by utilizing the power of nature. The prognosis and quality of life for people affected by oral cancer show considerable promise and scope for improvement with more study and development in this field.
